# Association of rs1570360 and rs2010963 in VEGF and rs2279744 in the MDM2 gene with Recurrent Implantation Failure in Iranian Women

**DOI:** 10.5935/1518-0557.20220062

**Published:** 2023

**Authors:** Mehdi Elmi, Pegah Ghandil, Masoud Hemadi, Maryam Tahmasebi Birgani, Alihossein Saberi

**Affiliations:** 1 Department of Medical Genetics, Faculty of Medicine, Ahvaz Jundishpur University of Medical sciences, Ahvaz, Iran; 2 Fertility, Infertility and Perinatology Research Center, Scholl of Medicine, Ahvaz Jundishpur University of Medical Sciences, Ahvaz, Iran

**Keywords:** recurrent implantation failure, polymorphisms, VEGF, MDM2

## Abstract

**Objective:**

The embryo implantation includes a complex sequence of signaling events, comprising numerous molecular mediators, such as ovarian hormones, cytokines, adhesion molecules and, growth factors. One of the critical factors in angiogenesis is the vascular endothelial growth factor (VEGF). The VEGF plays a pivotal role in embryonic development, decidua vascularization and placental angiogenesis. Furthermore, the P53 gene and its negative regulator, murine double minute 2 (MDM2), are major players in reproductive processes. This study aimed to assess the association of polymorphisms of the VEGF and the MDM2 genes with idiopathic recurrent implantation failure.

**Methods:**

We genotyped 60 women with previous idiopathic recurrent implantation failures and 60 fertile women as controls. Restriction Fragment Length Polymorphism (RFLP) and Sanger sequencing were used for genotyping the rs2010963 and the rs1570360 polymorphisms in VEGF; and the rs2279744 in MDM2 genes.

**Results:**

Results indicated a higher frequency of the VEGF rs1570360 AA genotype and A allele in patients with a history of idiopathic implantation failure [OR=6.4 (1.22 - 33.64), p-value=0.02)]. However, the frequency of VEGF +405 G/C and MDM2 SNP309 T/G [(OR=3 (0.5 - 16) p-value=0.2, OR=1.18 (0.3 - 3.7) p-value=0.7, respectively)] genotypes were not significantly different between cases and controls.

**Conclusions:**

The VEGF polymorphism may influence embryo implantation and the VEGF rs1570360 AA genotype may predispose to the risk of recurrent implantation failure after IVF.

## INTRODUCTION

Infertility has increased in recent decades, and the estimated prevalence of infertility is 10-15 percent in the general reproductive-age couple (Chan *et al*., 2016a; Zeng *et al*., 2017). Recurrent Implantation Failure (RIF) is considered to be a repetitive failure to achieve pregnancy following the transfer of high-quality embryos after several *in-vitro* fertilization (IVF) treatment attempts (Jung *et al*., 2016).

The causes of RIF are still poorly known. Its etiology can be divided into three categories: decreased endometrial receptivity, embryonic defects, and unsynchronized relation between maternal and embryonic tissue (Vagnini *et al*., 2015). Uterine abnormalities, including thin endometrium and the altered expression of cell adhesion molecules, may lead to a decrease in endometrial receptivity. Genetic abnormalities like chromosome anomalies and sperm defects play a vital role in RIF (Boudjenah *et al*., 2012). It is demonstrated that low expression of apoptosis and angiogenesis genes is related to implantation failure (Lledo *et al*., 2014).

The vascular endothelial growth factor (VEGF) is an essential factor in the angiogenesis and an initial regulator of endothelial cells proliferation. In the early gestation, VEGF is crucial for oocyte maturation, trophoblastic growth, implantation, and fetal, placental angiogenesis in the uterus (Xu *et al*., 2015). The gene which codes VEGF on chromosome 6p21.3 involves a 14kb coding region with eight exons and seven introns (Samli *et al*., 2012). Until now, seven isoforms of human VEGF have been identified (Galazios *et al*., 2009). The P53 gene has an essential role in fertility by regulating the expression of the leukemia inhibitory factor (LIF) gene, which plays a necessary role in the implantation process (Winship *et al*., 2018). Human orthologous of murine double minute 2 (MDM2) is one of the main negative regulators of P53 (Chan *et al*., 2016a). The MDM2 gene contains 12 exons and three promoters, which are located on the long arm of chromosome 12 in humans, with a length of 34 kb (Chan *et al*., 2016b).

Numerous studies have identified a variety of polymorphisms in the VEGF gene, some of these include -2576 A/C and -1154 G/A in the promoter region, and +405 G/C in the 5’UTR and regions of 936 C/T in the 3’UTR, associated with the alteration of VEGF expression (Sajjadi *et al*., 2020). Researchers also looked at the role of VEGF polymorphisms in embryonic implantation. For example, the association of -1154G/A polymorphism and repeated implantation failure after IVF has been reported (Boudjenah *et al*., 2012). The MDM2 SNP309 contains the T to G conversion in the regulatory region of the first intron. In SNP309, the G allele tends to increase the binding of the SP1 activator transcription factor to the DNA, which increases the level of MDM2 and decreases the p53 activity. Also, the SNP309 is located in an area that is directly regulated by estrogen signaling. Estrogen preferably stimulates the transcription of MDM2 with the SNP309G allele (Hu *et al*., 2011). To our knowledge, there is no study about the association of VEGF and MDM2 polymorphisms with RIF in Iranian women.

Because implantation is a complex process and there are many pathways involved, the study of one pathway is not responsive to repeated implantation failure. Therefore, two genes that are involved in different mechanisms were selected for this study, which purpose was to investigate the association between rs1570360 and rs2010963 polymorphisms in the VEGF gene and the rs2279744 polymorphism in the MDM2 gene in women with the risk of recurrent failures in IVF technique, for the first time in Iranian women.

## MATERIALS AND METHODS

### Study population

We had 120 women from Arab and Fars ethnicity included in this study (60 women with a history of recurrent implantation failure after IVF embryo transfer and 60 fertile women with at least one live birth served as controls). Written informed consent was obtained from all the participating women, and the Institutional Ethics Committee of the Ahvaz Jundishapour University of Medical Sciences confirmed the study (Ethics code: IR.AJUMS.REC.1396.572). RIF was defined in this study as a transfer of a cumulative total of four good quality embryos with less than 5 mIU/ml hCG serum levels 14 days after embryo transfer. All transferred embryos were considered to be of good quality by the embryologist. Patients with a history of autoimmune disease, abnormal uterine anatomy - based on a laparoscopic examination, ovarian cysts, endometriosis, and chromosome abnormalities were excluded from the study.

### DNA extraction and genotyping

DNA was isolated from whole blood samples (5 ml collected into EDTA tubes) taken from RIF patients and control subjects. The DNA extraction was performed by a DNA Extraction Kit (Favorgen Biotech, Taiwan) according to the manufacturer’s protocol. Genotyping was carried out by restriction fragment length polymorphism (RFLP) after polymerase chain reaction (PCR) and confirmed by Sanger sequencing. Supplementary Table 1 shows the detailed information about the PCR-RFLP procedure (Baitello *et al*., 2016; Avirmed *et al*., 2017; Papazoglou *et al*., 2005).

### Statistical analysis

The differences in genotype frequencies of the VEGF and MDM2 gene polymorphisms were analyzed using the Chi-square test. The association between these polymorphisms and RIF was calculated with 95% odds ratios (OR) and confidence intervals (CI). A *p*-value <0.05 was considered statistically significant. The Mann-Whitney U-test was used to analyze the patients’ characteristics. The Hardy-Weinberg equilibrium was tested in the RIF patients and controls separately. All statistical analyses were performed using the SPSS software version 22.0 (SPSS, Chicago, IL, USA). The haplotype frequencies were estimated using the Haploview 4.1.

## RESULTS

A total of 120 women including 60 women with a history of recurrent implantation failure after IVF embryo transfer (mean age ±SD = 31.72±4.71) and 60 fertile women with at least one live birth, served as controls (mean age ± SD = 31.91±6.3), were enrolled in this study. There was no significant difference between the cases and controls concerning the mean age (*p*-value=0.8). Also, the mean patients’ BMIs (26.5±4.06) were not significantly different (*p*-value=0.6) from the controls (26.8±4.6). The mean of recurrent implantation failure was 1.72±0.9 (mean±SD) in the patient group.

The genotype and allelic frequencies obtained in our study were consistent with the Hardy-Weinberg Equilibrium (HWE) for rs2010963 and rs2279744 in cases (*p*-value=0.005 and *p*-value=0.001, respectively) and controls (*p*-value=0.03 and 0.001, respectively) (Table 1). However, for the rs1570360, the genotype and allelic frequency were consistent with HWE only for the case group (*p*-value=0.001), while for the controls, it was not in equilibrium with the Hardy-Weinberg law (*p*-value=0.9). It is probably due to the small sample size and high consanguinity, especially Arab ethnicity, in our study population.

**Table 1:** Genotype and allelic frequencies of VEGF and MDM polymorphisms in recurrent implantation failure and control subjects.

Genotype / allele	Control n=60	Case n=60	OR (95%CI)	*p* value
rs1570360 GG AG AA G A HWE *p*-value	27 (45.0) 26 (43.3) 7 (11.7) 80 (66.7) 40 (33.3) 0.9	3 (5.0) 52 (86.7) 5 (8.3) 58 (48.3) 62 (51.7) 0.001	1 18 (4.99 - 64.89) 6.4 (1.22 - 33.64) 1 2.1 (1.26 - 3.60)	0.001 0.02 0.004
rs2010963 GG GC CC G C HWE *p*-value	6 (10.0) 44 (73.3) 10 (16.7) 56 (46.7) 64 (53.3) 0.03	2 (3.3) 45 (75.0) 13 (21.7) 49 (40.8) 71 (59.2) 0.005	1 3 (0.5 - 16) 0.6 (0.4 - 0.9) 1 0.3 (0.7 - 2.1)	0.2 0.9 0.3
rs2279744 TT TG T G HWE *p*-value	7 (11.7) 53 (88.3) 67 (55.8) 53 (44.2) 0.001	6 (10) 54 (90) 66 (55) 54 (45) 0.001	1 1.18 (0.3-3.7) 1 0.9 (0.5 - 1.6)	0.7 0.8

Table 1 also represents the distribution of the rs1570360, rs2010963, and rs2279744 genotypes and allelic frequencies for cases and controls.

The frequency of AA (wild type), AG and GG of VEGF rs1570360 was 8.3%, 86.7%, and 5% in patients, then 11.7%, 43.3% and 45% in controls, respectively. There was a significant association between the heterozygous AG genotype of VEGF rs1570360 gene polymorphism and RIF [OR=6.4 (1.22-33.64), *p*-value=0.001].

The genotypic frequencies of VEGF rs2010963 showed that CC, CG, and GG was 21.7%, 75%, and 3.3% in patients, on the other hand 16.7%, 73.3%, and 10% in controls, respectively. No significant association was found between VEGF rs2010963 gene polymorphism and the risk of RIF. However, the variant (CC and CG) genotype frequencies were more common in the cases as compared with controls. The frequency of the MDM2 rs2279744 TG genotype was more prevalent in the RIF group, but it was not statistically different [OR=1.18 (0.3-3.7), *p*-value=0.7] between controls and the RIF group.

We checked the genetic model of VEGF and MDM2 polymorphisms in RIF patients and control subjects to see which model is significantly associated with the risk of RIF (supplementary material Supplementary Table 2). The dominant model of inheritance showed significant association with variant VEGF rs1570360 genotypes [OR=15.5 (4.3 - 55.2), *p*-value=0.0001]; while the recessive model genotypes did not show any significant association [OR= 0.6(0.2 - 2.3), *p*-value=0.5]. The genotype of the VEGF rs2010963 polymorphism did not associate significantly with RIF under any of the inheritance models (Table 2). For the MDM2 rs2279744 TG genotype variant the dominant model of inheritance did not represent significant relationship with RIF [OR=0.8 (0.2-2.6), *p*-value=0.1].

**Table 2:** Haplotype frequencies of VEGF (rs1570360/rs2010963) polymorphisms in recurrent implantation failure.

ID	Control 2 n=120	RIF 2 n=120	OR (95%CI)	*p*-value
G-C	59 (49.16)	53 (44.16)	0.818 (0.492-1.359)	0.4
A-G	35 (29.16)	44 (36.6)	1.406 (0.818-2.415)	0.2
G-G	21 (17.5)	5 (4.16)	0.205 (0.075-0.564)	0.0008
A-C	5 (4.16)	18 (15)	4.059 (1.455-11.324)	0.004

To check, whether there was a synergistic effect of polymorphic site interactions on the risk of RIF, we analyzed the allele combination of the two VEGF polymorphisms (Table 2). Our analyses indicated that rs1570369/rs2010963 G-G and A-C haplotypes applied synergistic effect on an elevated risk of RIF [RIF (OR=0.2 (0.075 - 0.56), *p*-value=0.00089, OR=4.06 (1.45 - 11.32), *p*-value=0.004, respectively].

## DISCUSSION

This investigation deciphered the possible association of MDM2 (rs2279744) and VEGF (rs2010963, rs1570360) polymorphisms with RIF development in Iranian women. The genotype and allelic frequency, which is observed only in the polymorphism of the -1154 G/A VEGF gene, demonstrated a significant difference. These data suggested that the presence of the A allele is associated with RIF. However, the analysis of the rs2010963 and rs2279744 in VEGF and MDM2 gene polymorphisms respectively, did not reveal any significant difference between genotypes in the RIF and control groups.

In this study, we think that VEGF and MDM2 polymorphisms are confined to endometrial factors for implantation failure because patients with uterine anomaly and related medical diseases were excluded, and high-quality embryos were selected for implantation.

Previous reports showed that VEGF concentration increased during the late secretory and pre-menstrual phases (Sugino *et al*., 2002). Kong *et al*. (2008) reported that the rs1570360 (-1154 G/A) polymorphism affected the serum VEGF level marginally. Goodman *et al*. (2008) showed that in females who are experiencing recurrent implant failure VEGF rs1570360 (-1154A /A) has been observed more frequently than in fertile females. Therefore, a “low expression” genotype such as VEGF rs1570360 (-1154G/A) would lead to the low concentration of the VEGF protein, which leads to lower angiogenesis, cytotrophoblast invasion and survival in implant failure. Because the blastocyst was shown at the implantation time as a source of VEGF, the correlation between RIF and partner genotypes would be interesting to examine. Indeed, the reduction in endometrial VEGF could be modulated by a G/G genotype in the partner because all embryos would be A/G genotype. Conversely, an increase in RIF might be seen with A/A genotype partners. Moreover, the authors demonstrated that Polymorphisms of the VEGF gene had been correlated with variation in VEGF protein production (Goodman *et al*., 2008; Kang *et al*., 2009). Taken together, these data suggest the role of VEGF in angiogenesis and endometrial decidualization, which is essential for a successful pregnancy. In contrast to our finding, -1154A/G polymorphism in the VEGF gene had an insignificant association with increased risk of RIF in the Brazilian population, according to Vagnini *et al*. (2015).

Genotype frequency of VEGF rs2010963 between RIF patients and controls was not significantly different (*p*-value=0.7). Conversely, in a study, Alidadiani & Salehi (2017) showed a higher frequency of the VEGF +405 GG genotype and the G allele in a patient with a history of IVF-ET failure. Also, Boudjenah *et al*. (2012) reported a higher frequency in RIF subjects after ICSI-ET than in controls. Inconsistent with our result, a study in the Arab population showed that the serum VEGF concentration was not affected by rs2010963 genotypes (Al-Habboubi *et al*., 2011). The controversy between different studies indicated that the VEGF serum level depends on population and ethnicity.

Based on the effects of genotype and allele frequency of MDM2 rs2279744 on missed abortion and IVF failure (Chan *et al*., 2016a; Fang *et al*., 2009), we checked to see the association of this polymorphism with RIF. Our analysis revealed no significant difference between RIF and control subjects, which is consistent with Ying Chan et al. The outcomes of some investigation about the association of VEGF rs2010963, MDM2 rs2279744 polymorphisms with recurrent implantation failure risk have been contradictory. For example, two recent studies indicated an association with increased risk of RIF (Kang *et al*., 2009; Alidadiani & Salehi, 2017), while studies in Bulgaria and China found no association with RIF risk (Chan *et al*., 2016a; Fang *et al*., 2009). Further studies with a large sample size across diverse ethnic communities should be performed to elucidate the association between rs1570360, rs2010963, and rs2279744 (MDM2 SNP309 T/G) polymorphisms and RIF in the Iranian population.

## CONCLUSION

In general, this is the first study, which investigates the association between rs1570360, rs2010963, and rs2279744 and the prevalence of RIF in the Iranian population. The findings obtained in the present study demonstrated that the VEGF rs1570360 polymorphism is associated with the risk of RIF in the studied population. In particular, the distribution of A allele was significantly higher in RIF cases than controls and it might affect the pathogenesis of recurrent implantation failure. This polymorphism may be useful biomarker for predicting individual susceptibility to RIF. We did not find significant association between rs2010963, and rs2279744 polymorphisms and RIF. Considering the limitations mentioned above, further studies are required to verify the functional significance of the VEGF - 1154A allele and how it participates in the pathogenesis of RIF

## Appendix

**Supplementary Table 1:** Details of PCR and RFLP procedures and observed products.

Genotype	Primers (F: forward and R:reverse)	PCR conditions	PCR product
**rs2010963**	F: 5´-ATTTATTTTTGCTTGCCA-3´ R: 5´-GTCTGTCTGTCTGTCCGTCA-3´	30 cycles: 95ºC 30 second, 53 ºC 30 second, 72ºC 30 second	304 bp
**rs1570360**	F5'- TCCTGCTCCCTCCTCGCCAATG-3' R5'-GGCGGGGACAGGCGAGCATC-3'	35 cycles: 94ºC 45 second, 62ºC 45 second, 72ºC 45 second	206 bp
**rs2279744**	F: 5′-CGG GAG TTC AGG GTA AAG GT-3′ R: 5′-AGCAAGTCGGTGCTTACCTG-3′	30 cycles: 94ºC 30 second, 58ºC 30 second, 72ºC 30 second	352 bp

* RE: restriction enzyme.

**Supplementary Table 2:** Genetic model of *VEGF* and *MDM2* polymorphisms in recurrent implantation failure patients and control subjects.

Genetic Model	Control n=60	Case n=60	OR (95%CI)	*p*-value
**rs1570360** **Dominant** **AA+AG** **GG** **Recessive** **AA** **GG+AG**	33 (55.0) 27 (45.0) 5 (8.3) 55 (91.7)	57 (95.0) 3 (5.0) 7 (11.7) 53 (88.3)	1 15.5 (4.3 - 55.2) 1 0.6 (0.2 - 2.3)	0.0001 0.5
**rs2010963** **Dominant** **CC+GC** **GG** **Recessive** **CC** **GG+GC**	54 (90.0) 6 (10.0) 10 (16.7) 50 (83.3)	58 (96.7) 2 (3.3) 13 (21.7) 47 (78.3)	1 3.2 (0.6 - 16.6) 1 1.3 (0.5 - 3.4)	0.1 0.4
**rs2279744** **Dominant** **GG+TG** **TT** **Recessive** **GG** **TT+TG**	53 (88.3) 7 (11.7) 0 60 (50.0)	54 (90) 6 (10) 0 60 (50.0)	1 0.8 (0.2-2.6) 1 0.9 (0.5 - 1.6)	0.7 NA

NA = not applicable.
